# The Probiotic Mixture VSL#3 Alters the Morphology and Secretion Profile of Both Polarized and Unpolarized Human Macrophages in a Polarization-Dependent Manner

**DOI:** 10.4172/2155-9899.1000227

**Published:** 2014-06-20

**Authors:** Raymond A. Isidro, Fernando J. Bonilla, Hendrick Pagan, Myrella L. Cruz, Pablo Lopez, Lenin Godoy, Siomara Hernandez, Raisa Y. Loucil-Alicea, Vanessa Rivera-Amill, Yasuhiro Yamamura, Angel A. Isidro, Caroline B. Appleyard

**Affiliations:** 1 Department of Physiology and Pharmacology, Ponce School of Medicine and Health Sciences, Ponce, PR 00716, USA; 2 Department of Microbiology, Ponce School of Medicine and Health Sciences, Ponce, PR 00716, USA; 3 Department of Biochemistry, Ponce School of Medicine and Health Sciences, Ponce, PR 00716, USA; 4 Department of Pathology, Ponce School of Medicine and Health Sciences, Ponce, PR 00716, USA

**Keywords:** M1, M2, macrophage, Inflammatory bowel disease, Ulcerative colitis, Crohn’s disease, Probiotic, VSL#3

## Abstract

**Background:**

Patients with Inflammatory Bowel Disease (IBD), most commonly Crohn’s disease (CD) or ulcerative colitis (UC), suffer from chronic intestinal inflammation of unknown etiology. Increased proinflammatory macrophages (M1) have been documented in tissue from patients with CD. Anti-inflammatory macrophages (M2) may play a role in UC given the preponderance of Th2 cytokines in this variant of IBD. Animal and clinical studies have shown that the probiotic VSL#3 can ameliorate signs and symptoms of IBD. Although animal data suggests a modulatory effect on macrophage phenotype, the effect of VSL#3 on human macrophages remains unknown.

**Objective:**

To determine the effect of the probiotic VSL#3 on the phenotype of polarized (M1/M2) and unpolarized (MΦ) human macrophages.

**Methods:**

Human monocyte-derived macrophages, generated by culturing monocytes with M-CSF, were left unpolarized or were polarized towards an M1 or an M2 phenotype by culture with LPS and IFN-γ or IL-4, respectively, and were then cultured in the presence or absence of VSL#3 for 3 days. Changes in macrophage morphology were assessed. Cytokine and chemokine levels in supernatants were determined by multiplex assay.

**Results:**

VSL#3 decreased the granuloma-like aggregates of M1 macrophages, increased fibroblast-like M2 macrophages, and decreased fibroblast-like MΦ macrophages. VSL#3 increased the secretion of IL-1β, IL-6, IL-10, and G-CSF by M1, M2, and MΦ macrophages. VSL#3 exposure maintained the proinflammatory phenotype of M1 macrophages, sustaining IL-12 secretion, increasing IL-23 secretion, and decreasing MDC secretion. Both VSL#3-treated M2 and MΦ macrophages secreted higher levels of anti-inflammatory and pro-healing factors such as IL-1Ra, IL-13, EGF, FGF-2, TGF-α, and VEGF, as well as proinflammatory cytokines, including IL-12 and TNF-α.

**Conclusion:**

Under our experimental conditions VSL#3 induced a mixed proinflammatory and anti-inflammatory phenotype in polarized and unpolarized macrophages. This differential effect could explain why patients with CD do not respond to probiotic therapy as well as patients with UC.

## Introduction

According to the Centers for Disease Control, over a million people in the United States alone are affected by Inflammatory Bowel Diseases (IBD), such as Crohn’s disease (CD) and ulcerative colitis (UC). The inflammation in IBD has been generally regarded as mediated by a type 1 helper T (Th1) cell response for CD and a type 2 helper T (Th2) cell response for UC [1]. The colon, or large intestine, is one of the gastrointestinal organs most affected by IBD and is one of the most macrophage- and bacteria-dense organs in the body [2,3]. While it remains unclear whether the altered microflora, or dysbiosis, seen in patients with IBD results from or causes the condition, several animal and clinical studies have shown that the pathology in animal models and patients can be improved by treatment with probiotics [4,5]. The Food and Agriculture Organization of the United Nations (FAO) and the World Health Organization (WHO) have defined probiotics as “live microorganisms which when administered in adequate amounts confer a health benefit to the host” (FAO/WHO, 2001). The probiotic mixture VSL#3 contains the following 8 gram-positive bacteria: *Bifidobacterium breve, Bifidobacterium infantis, Bifidobacterium longum, Lactobacillus acidophilus, Lactobacillus delbrueckii* subspecies *bulgaricus, Lactobacillus casei, Lactobacillus plantarum,* and *Streptococcus salivarius* subspecies *thermophilus.* VSL#3 has been shown to ameliorate inflammation in murine models of IBD [6] and improve signs and symptoms of IBD in patients [7–9].

Recent findings by Bassaganya-Riera et al. in a mouse model of acute DSS colitis suggest that VSL#3 acts by influencing macrophage phenotype, specifically, by reducing the proportion of colonic proinflammatory macrophages [10]. Previous studies have evaluated the direct effect of VSL#3 on monocyte-derived dendritic cells [11–13], but the effect that this particular probiotic formulation has on monocyte-derived macrophages and/or human macrophages has not been studied. Macrophages are a heterogeneous group of mononuclear cells that play key roles in the defense and repair of the host [14]. These cells can be functionally and phenotypically classified into macrophages that either promote (classically-activated macrophages, M1) or antagonize (alternatively-activated macrophages, M2) inflammation, somewhat analogous to the Th1–Th2 dichotomy of helper T cells [15,16]. Kamada et al. reported an increased presence of M1 macrophages in CD [17]. Notably, M2 macrophages result from stimulation with Th2 cytokines, such as IL-4 and IL-13 [18–23], and thus may contribute to the pathogenesis of UC [23].

In the present study, we used M1 and M2 human monocyte-derived macrophages as a model system of macrophages present in patients with CD and UC, respectively, and sought to determine the effect of the probiotic mixture VSL#3 on these cells, as well as on unpolarized macrophages (MΦ). We tested the hypothesis that the probiotic would lead these macrophages to decrease proinflammatory cytokine and chemokine secretion and increase secretion of anti-inflammatory cytokines and chemokines, regardless of polarization status. Curiously, we found that M1, M2, and MΦ macrophages exposed to VSL#3 secreted higher levels of certain proinflammatory and anti-inflammatory factors and lower levels of others. We also found that exposure to the probiotic VSL#3 produced marked alterations in the morphology of M1, M2, and MΦ macrophages. Of note, both the secretory and morphological changes were largely dependent on the initial macrophage polarization status.

## Materials and Methods

### Ethics statement

All studies discussed herein were approved by the Institutional Review Board (FWA 00000345) at Ponce School of Medicine and Health Sciences (Ponce, PR, USA). Participating volunteers gave informed consent.

### Monocyte isolation

Blood from healthy volunteers [17,24,25] was collected in plastic EDTA blood collection tubes. Peripheral blood mononuclear cells (PBMCs) from blood diluted ~1:3.5 in (PBS + 2 mM EDTA) were obtained by density gradient centrifugation with Ficoll-Paque Premium (GE Healthcare Life Sciences, Pittsburgh, PA, USA) and pooled in equal parts in order to obtain an equal representation from each volunteer [24,25]. Monocytes were isolated from pooled PBMCs by positive selection using CD14 magnetic microbeads (Miltenyi Biotec Inc., Auburn, CA, USA), according to the manufacturer’s instructions, and purity was confirmed by flow cytometry.

### Macrophage culture and morphological analysis

**Figure 1:**
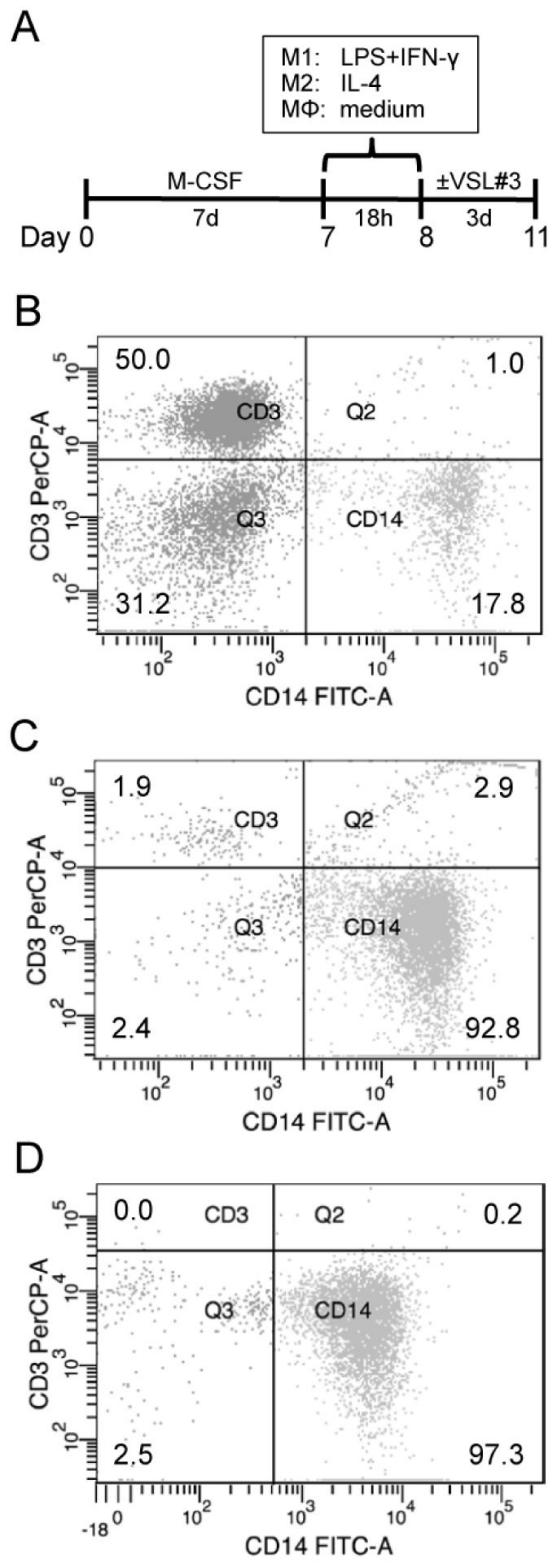
Experimental design and purity isolated cells. (A) Monocytes isolated from the blood of healthy donors were differentiated into macrophages by 7-day culture with M-CSF, and the resulting macrophages were cultured for 18 hours with polarizing factors or medium and then for three days with or without the probiotic VSL#3. (B–D) PBMC and monocyte samples were taken on day 0 after Ficoll-Paque centrifugation and magnetic bead purification, respectively, while the macrophage sample was taken from adhered cells on day 7. Cells were stained with anti-CD3-PerCP and anti-CD14-FITC antibodies and analyzed on a FACSAria flow cytometer. Representative graphs are shown.

Polarized monocyte-derived macrophages were generated by a modification of the method by Martinez et al. [18–23]. On day 0 (Figure 1A), monocytes were plated in 6-well plates at a density of 5×10^5^ cells/mL in 3 mL of RPMI 1640 medium (Thermo Scientific, Waltham, MA, USA) supplemented with 20% heat-inactivated fetal bovine serum (FBS; Thermo Scientific), 100 U/mL of penicillin, 100 μg/mL streptomycin, and 100 ng/mL macrophage colony-stimulating factor (M-CSF; Peprotech, Rocky Hill, NJ, USA). The medium was removed on day 7, and replaced with fresh RPMI containing polarizing factors and supplemented with 5% heat-inactivated FBS, 100 U/mL penicillin, 100 μg/mL streptomycin. For M1 macrophages, polarizing factors consisted of 20 ng/mL interferon (IFN)-γ (Peprotech) and 100 ng/mL lipopolysaccharide (LPS; Sigma-Aldrich, St. Louis, MO, USA), and, for M2 macrophages, 20 ng/mL interleukin (IL)-4 (Peprotech). Unpolarized (MΦ) macrophages were incubated in medium free of polarizing factors. After 18 hours (day 8), a 0.5 mL sample of supernatant was collected to establish baseline cytokine levels and confirm polarization status. Then, 0.5 mL of fresh medium was added, and macrophages were cultured for an additional three days in the absence or presence of 3.33×10^7^ colony-forming units (CFU) of the probiotic mixture VSL#3 (VSL Pharmaceuticals, Gaithersburg, MD, USA) [11] dissolved in phosphate-buffered saline (PBS). Controls received PBS only. The center and periphery of each well was photographed to remove any potential for bias due to differences in distribution, and supernatant from each well was collected on day 11. Streak plates were performed for the probiotic preparation before addition on day 8 and for the supernatants on day 11. The growth of several colonies on the plate for the probiotic preparation and of none on the plate for the supernatants from day 11 indicates a reduction in bacterial viability by the end of the three-day incubation period. This reduction in bacterial viability was to be expected given that the culture medium contained antibiotic and that macrophages can avidly phagocytize bacteria [26]. Fibroblast-like, round/oval (loose or in clusters), and total cells per mm^2^ were quantified and fibroblast-like cell length was measured with ImageJ v1.47m (NIH, Bethesda, MD, USA).

### Flow cytometry

Monocyte and macrophage purity was assessed on day 0 and day 7, respectively, by staining cells with anti-CD14-FITC and anti-CD3-PerCP antibodies. IgG-FITC and IgG-PerCP were used as isotype controls, and all antibodies were from BD Biosciences (San Jose, CA, USA). Cells were incubated with antibody in the dark for 30 minutes, washed with staining buffer (PBS, 1% FBS, 0.1% sodium azide), and fixed with 0.5% paraformaldehyde. Stained cells were analyzed with a FACSAria flow cytometer (BD, Franklin Lakes, NJ, USA).

### Cytokine measurement and analysis

The levels of IL-1β, IL-4, IL-6, IL-10, IL-12p70, IL-23, and TNF-α in supernatants collected on days 8 and 11 were measured by multiplex assay (HTH17MAG-14K-07, EMD Millipore, Billerica, MA, USA). A second multiplex assay (HCYTMAG-60K-PX38, EMD Millipore) was used to measure levels of 38 cytokines and chemokines. For each sample, the cytokine levels at day 8 were subtracted from those at day 11 to obtain the change in cytokine and chemokine secretion (ΔCCS). The fold change in the mean ΔCCS between untreated and VSL#3-treated macrophages was computed for each type of macrophage by dividing the mean ΔCCS of VSL#3-treated macrophages by the mean ΔCCS of untreated macrophages.

### Statistical analysis

Differences in morphology between treatment groups were compared via one-way ANOVA with a Tukey’s multiple comparisons test in Prism v6.0a (Graphpad Software, Inc., La Jolla, CA, USA). A *P* value less than 0.05 was considered statistically significant. For cytokine analyses, descriptive statistics were computed with SPSS v21 (IBM Corp., Armonk, NY, USA) and a fold change in mean ΔCCS of ≤2 or ≥0.5 was deemed significant only if the 95% confidence intervals for the mean ΔCCS of the two treatments did not overlap. A fold change of ≤0.5 indicates that the mean ΔCCS of VSL#3-treated macrophages was half or less than the mean ΔCCS of untreated macrophages, while a fold change of ≥2 indicates that the mean ΔCCS of VSL#3-treated macrophages was twice or more than the mean ΔCCS of untreated macrophages.

### Online supplemental material

For cytokines appearing in Tables 1–3, the mean levels ± standard error of the mean (SEM) on days 8 and 11 are provided in Supplemental Tables 1–3, respectively.

## Results and Discussion

### Purity of isolated cells

The percentage of cells positive for the monocyte/macrophage marker CD14 and the T lymphocyte marker CD3 in PBMCs obtained by density gradient centrifugation (Figure 1B), monocytes isolated using CD14 microbeads (Figure 1C), and adherent monocyte-derived macrophages (Figure 1D) was determined by flow cytometry. The percentage of CD14^+^ cells increased while the percentage of CD3^+^ cells decreased with each purification step. PBMCs were ~50% CD3^+^ and ≤20% CD14^+^, isolated monocytes were < 3% CD3^+^ and ≥92% CD14^+^, and monocyte-derived macrophages were 0% CD3^+^ and > 97% CD14^+^. Forward and side-scatter analysis indicates that the CD14^-^cells in samples from monocyte-derived macrophages have properties consistent with dead cells and/or debris. We are therefore confident that our subsequent observations result in fact from the behavior of macrophages and not from other contaminating PBMCs.

### Effect of VSL#3 on macrophage morphology

**Figure 2:**
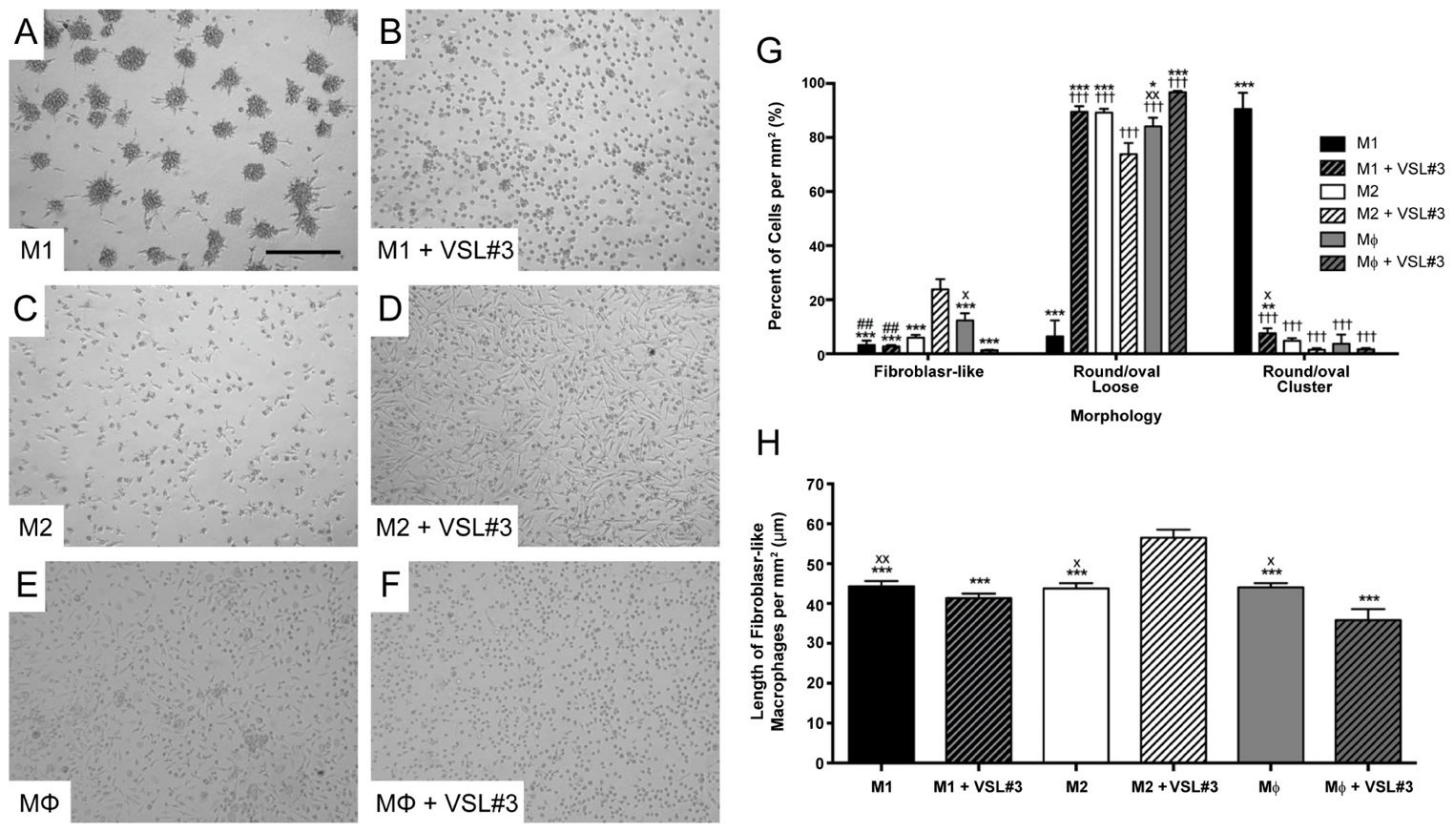
VSL#3-induced changes in macrophage morphology. (A–D) Representative micrographs illustrating the morphology of untreated (A,C,E) and treated (B,D,F) M1, M2, and MΦ macrophages at day 11 of the protocol. Treatment consisted of exposure to 3.33x107 CFU of the probiotic mixture VSL#3 for 3 days. Scale bar = 0.25 mm in A and applies to A–F. (G) Quantification of cells demonstrating different morphological characteristics. Cells were quantified by using the ‘cell counter’ and ‘analyze particles’ features of Image J. Two high-powered fields (mm^2^) were counted per well, one in the center of the well and another in the periphery. Data represents the mean ± SE of 10–12 counts per treatment, half in the center and half in the periphery. (H) Length of the fibroblast-like macrophages quantified in G, measured with ImageJ. Data were analyzed using one-way ANOVA and Tukey’s multiple comparisons test. *p<0.05, ***p<0.001 vs M2+VSL#3; ^##^p<0.01 vs MΦ; ^†††^p<0.001 vs M1, ^x^p<0.05, ^xx^p<0.01 vs MΦ+VSL#3.

Exposure to VSL#3 led to pronounced morphological changes in M1, M2, and MΦ macrophages, which largely depended on the initial polarization status (Figure 2). Exposure of M1 macrophages, which under our experimental conditions formed granuloma-like aggregates of round/oval cells with few fibroblast-like and loose round/oval cells, to VSL#3 significantly increased the percentage of loose round/oval cells and decreased the percentage of round/oval cells in clusters without changing the percentage of fibroblast-like cells (Figures 2A,2B, 2G). For M2 macrophages, treatment with VSL#3 significantly increased the proportion of fibroblast-like cells (p<0.001), which was the largest observed for this morphological subset, and significantly decreased proportion of loose round/oval cells (p<0.001) when compared to untreated M2 macrophages (Figures 2C,2D,2G). Additionally, VSL#3 led to a significant increase in the length of M2 macrophages with a fibroblast-like morphology (p<0.001; Figure 2H). Treating MΦ macrophages with VSL#3 led to a significant increase in loose round/oval cells (p<0.01) with a concomitant reduction in the percentage (p<0.05) and length (p<0.05) of fibroblast-like cells as compared to untreated MΦ macrophages (Figure 2E–2H). Few reports have described the morphology of *in vitro*-derived M1 and M2 macrophages. Rey-Giraud et al. found morphological characteristics similar to ours for macrophages generated by 6-day culture in RPMI with 10% FBS and either GM-CSF (M1) or M-CSF (M2): M1 macrophages were mostly round and oval cells and M2 macrophages were mostly fibroblast-like [27]. Edin et al. also found similar patterns when using conditions more akin to those used in the present study [28]. Neither of these two studies quantified the morphological subtypes of cells, making our study the first to do so. Our study is also the first to evaluate the effect of a probiotic on the morphology of macrophages. McWhorter et al. described a relationship between macrophage morphology and phenotype in which increasing elongation augments M2 phenotype [29], suggesting that our observed VSL#3-induced morphological changes in macrophages may indicate phenotypic alterations. In light of the studies by Rey-Giraud et al., Edin et al., and McWhorter et al., our observed changes seem to suggest the following: first, that the proinflammatory phenotype of M1 macrophages seems relatively unchanged by VSL#3 as the cells maintained a round/oval shape, even when changing from a clustered to a loose arrangement; second, that the anti-inflammatory phenotype of M2 macrophages is augmented by VSL#3 given that the probiotic increased both the number and length of cells with a fibroblast-like shape; and third, that MΦ were turned proinflammatory by exposure to VSL#3. To confirm whether these phenotypic alterations were indeed manifested by the different macrophages, we next measured cytokine and chemokine secretion in supernatants.

### Effect of VSL#3 on macrophage cytokine and chemokine secretion

**Figure 3:**
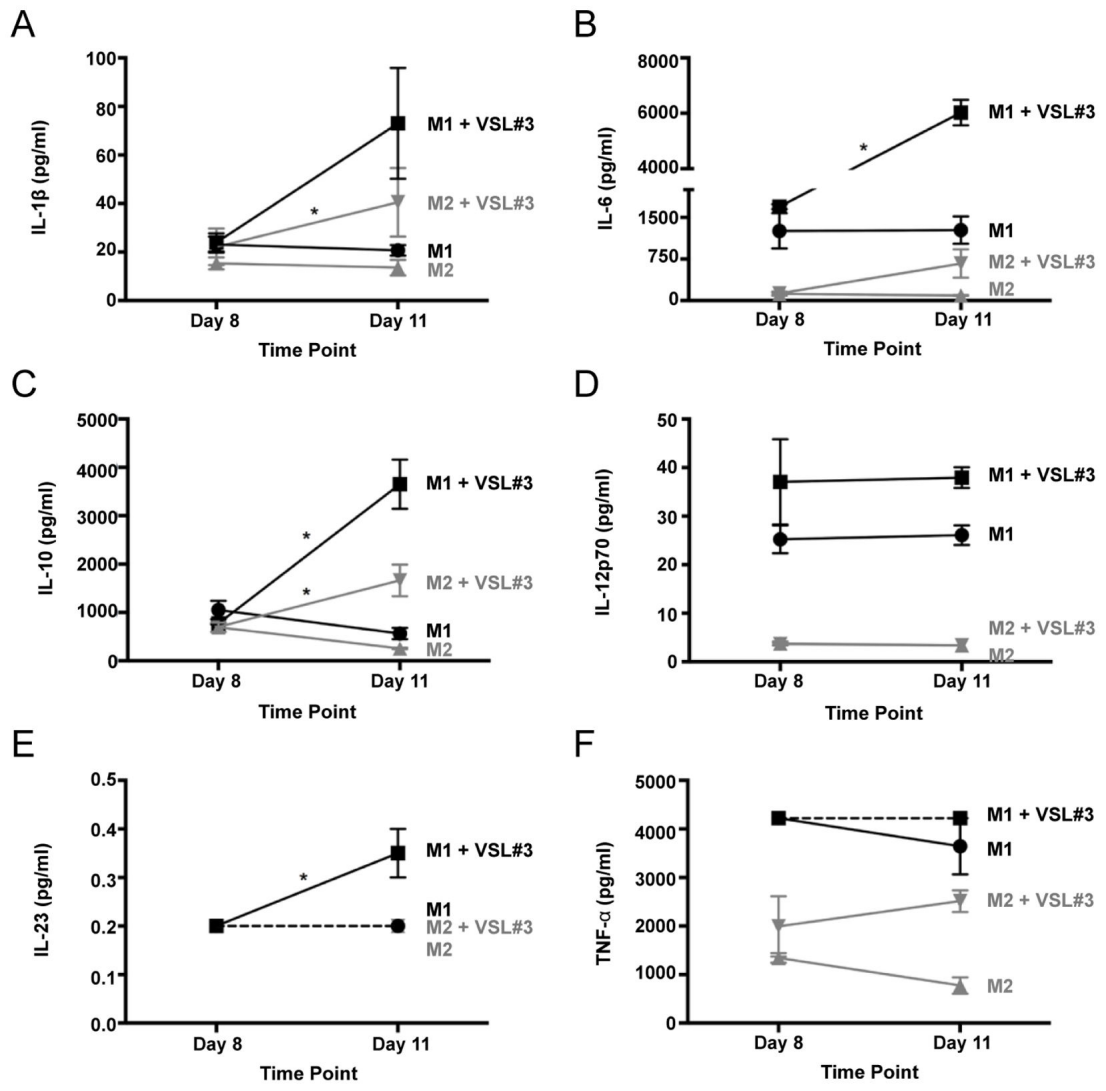
VSL#3-induced changes in cytokine secretion by M1 and M2 macrophages. Levels of IL-1β (A), IL-6 (B), IL-10 (C), IL-12p70 (D), IL-23 (E), and TNF-α (F) were measured using a multiplex assay. Samples of supernatant were taken after 18-hour exposure to polarizing factors (day 8) and subsequent 3-day culture in the presence or absence of the probiotic mixture VSL#3 (day 11). Dashed lines indicate that levels were below or above the limits of detection. Each data point represents the mean ± SE of two pooled samples. * denotes a statistically significant difference in the mean change in cytokine and chemokine secretion (ΔCCS) of the treated macrophage vs. the untreated macrophage type, calculated as indicated in the methods section.

VSL#3 exposure also produced profound polarization-dependent changes in cytokine and chemokine secretion by macrophages. We first set out to determine the effect of VSL#3 on M1 and M2 macrophage secretion of the acute-phase cytokines IL-1β, IL-6, and TNF-α, the M1 cytokines IL-12p70 and IL-23, and the M2 cytokines IL-4 and IL-10 by way of a 7-plex assay (Figure 3). M1 macrophages exposed to VSL#3 secreted significantly higher levels of IL-6, IL-10, and IL-23 than their untreated counterparts (Figures 3B,3C,3E). Notably, levels of IL-12p70 did not change in response to the probiotic (Figure 3D). IL-4 was not detected in supernatant from any M1 macrophages, and its levels exceeded the limits of detection for M2 macrophages (data not shown). VSL#3 exposure increased M2 macrophage secretion of IL-1β and IL-10 (Figure 3A and 3C).

After establishing that VSL#3 induced changes in the secretion of certain cytokines by polarized macrophages, we next wanted to better characterize the effect of VSL#3 on the secretion profile of both polarized and unpolarized macrophages. For this, we employed a multiplex assay to assess VSL#3-induced changes on the levels of 38 cytokines and chemokines. The quality control values for 3 of the 38 cytokines were outside of the appropriate range and were thus excluded from any further analysis. Our analyses revealed that VSL#3 significantly altered the secretion of 32 cytokines by the three macrophage types under study. The cytokines with significant changes in secretion, as determined by the described method, are shown for M1, M2, and MΦ macrophages in Tables 1–3, respectively. The levels on days 8 and 11 for cytokines in Tables 1–3 are listed in Supplemental Tables 1–3, respectively. All three types of macrophages secreted higher levels of IL-1β, IL-6, and G-CSF in response to VSL#3. Changes in the secretion of the remaining cytokines/chemokines were exclusive to either M1, M2, or MΦ macrophages or a combination therein. Therefore, changes for the majority of the cytokines/chemokines were dependent on the macrophages’ initial polarization state. Only treated M1 macrophages increased secretion of IL-1α and IL-4 and decreased secretion of IL-17A. Increased fractalkine secretion was seen only in VSL#3-treated M2 macrophages. MΦ macrophages were the only ones to secrete higher levels of GM-CSF and IFN-γ and lower levels of MCP-1 in response to VSL#3. Treatment with the probiotic decreased MDC secretion by both M1 and MΦ macrophages. Expression changes of the remaining 21 cytokines were seen in M2 and MΦ macrophages. Of note, increased secretion of the M2 cytokines IL-1Ra, IL-10, and IL-13 and the pro-healing factors EGF, FGF-2, TGF-α, and VEGF was observed for both M2 and MΦ macrophages. These two types of macrophages also secreted higher levels of the pro-inflammatory cytokines IL-12 (p40 & p70) and TNF-α and chemokines IL-8, eotaxin, GRO, and MIP-1 (α & β) after exposure to VSL#3.

Overall, the baseline cytokine and chemokine secretion patterns we obtained for human M1 and M2 macrophages were consistent with those previously reported by Rey-Giraud et al. [27]. Of the 30 analytes reported in their supplementary data, 24 coincided with those measured in our study. For those analytes that coincided, secretion patterns were discordant only for EGF, IL-1Ra, and TGF-α for which we found higher secretion by M1 macrophages, but Rey-Giraud et al. found higher secretion by M2 macrophages. We find it interesting that in both studies M1 macrophage secretion of IL-10 and VEGF was higher than that of M2 macrophages, especially since these two soluble factors are generally regarded as characteristic for M2 macrophages. Although some level of IL-10 secretion is expected in M1 macrophages as a result of NF-κB activation [30], it is nevertheless surprising that IL-10 secretion in M1 macrophages surpasses that of M2 macrophages.

Several studies have examined the effect of VSL#3 on dendritic cell secretion of IL-10 and IL-12p70 [11–13]. Hart et al. reported intracellular levels of IL-10 to be increased and of IL-12p70 to be decreased in human dendritic cells from peripheral blood and intestinal lamina propria [13]. Studies by Drakes et al. in mice bone marrow-derived dendritic cells showed that VSL#3 induced higher levels of IL-10 and these greatly surpassed levels of IL-12p70, which were increased within the first day of culture but subsequently decreased [11]. Finally, Gad et al. reported that various concentrations of VSL#3 led to higher secretion of IL-12p70 than IL-10 within a 24-hour period, but they did not look at later time points [12]. Gut bacteria interact directly with macrophages given that the gut microflora that normally penetrate the epithelial barrier are cleared mostly by macrophages [26] and that resident intestinal macrophages have been shown to extend transepithelial dendrites into the lumen of the gut [31,32] and to contribute to oral tolerance by transferring antigens to dendritic cells through gap junctions [33]. Therefore, we decided to examine the direct effect of VSL#3 on macrophages.

We chose to examine VSL#3 induced changes on cytokine secretion after a three-day incubation for several reasons. First, we believed that changes occurring over several days might be more representative of the events that unfold when probiotics are ingested. Second, we reasoned that leukocyte behavior 72 hours after stimulation would be more informative in terms of an immune response because this is the time period that is generally regarded as necessary for an adaptive immune response to take place. Third, it takes days rather than hours for signaling-induced changes in transcription to result in altered protein synthesis and secretion.

Exposure to the probiotic led to a mixed phenotype whose tendency towards inflammation depended on the macrophages’ polarization status. VSL#3 did not induce a strictly proinflammatory or anti-inflammatory response in polarized and unpolarized macrophages, contrary to our hypothesis. VSL#3-exposed M1 macrophages maintained a predominantly proinflammatory phenotype as evidenced by sustained levels of IL-12 secretion and increased secretion of inflammatory cytokines such as IL-1β, IL-6, IL-23, and G-CSF. While IL-10 was over the detection limit in the second multiplex, the results from the first multiplex clearly show that IL-10 secretion by M1 macrophages increased upon treatment with VSL#3. The fact that levels of TNF-α for M1 macrophages exceeded the limits of detection of both multiplex assays regardless of VSL#3 treatment confirms that these macrophages are indeed proinflammatory. On the other hand, M2 and MΦ macrophages exposed to VSL#3 secrete increased levels of anti-inflammatory and pro-healing factors in addition to the inflammatory factors, thus seemingly adopting a more balanced phenotype. The differential effect of VSL#3 on M1 and M2 macrophages could perhaps explain why VSL#3 and other probiotics have been more effective to date in patients with UC than in patients with CD [4,5]. The increase in proinflammatory cytokines might aggravate inflammation in CD by fueling the Th1 response, but might improve the condition for UC patients by shifting the balance away from a Th2 response. The hybrid phenotype induced in M2 and MΦ macrophages by VSL#3 resembles the regulatory, or type II, (M2b) macrophage, a subtype of M2 macrophages characterized by secretion of high levels of the anti-inflammatory cytokine IL-10 and the pro-inflammatory cytokines IL-1β, IL-6, and TNF-α, while secreting lower levels of IL-12 [15]. Although this type of macrophage was initially thought to result from stimulation with immune complexes and toll-like receptor ligands, recent reports indicate that M2b macrophages may also be induced by inflammatory clearance of apoptotic neutrophils [34] and dectin-1 activation by ligands such as zymozan [35]. Further investigation is needed to determine if probiotics and possibly commensal flora can also trigger an M2b phenotype.

In conclusion, we have shown for the first time the direct effect of VSL#3 on human macrophage morphology and secretion. Specifically, our study demonstrates that VSL#3 has distinct effects on both polarized M1 and M2 and unpolarized MΦ macrophages. Our results emphasize the need for studying the effects of potential therapeutic strategies for IBD in the context of the two main forms of this condition, UC and CD.

## Supplementary Material

All supplementary tables

## Figures and Tables

**Table 1:** Cytokines differentially secreted by M1 macrophages in response to VSL#3.

**Analyte**	**M1 (pg/mL)**		**M1 + VSL#3 (pg/mL)**		**Fold-Change**
	**Mean ΔCCS**	**95% CI**	**Mean ΔCCS**	**95% CI**	35.75
IL-1α #	−0.40	−0.40, −0.40	13.90	11.74, 16.06	
IL-1β *	−39.95	−71.41, −8.49	324.90	277.47, 372.33	9.13
IL-4 #	−3.95	−4.24, −3.66	8.40	3.89, 12.91	3.13
IL-6 *	−1060.76	−2214.73, 93.21	5902.52	3883.72, 7921.32	6.56
IL-17A #	−1.80	−3.17, −0.43	−4.05	−4.34, −3.76	2.25
G-CSF *	−95.50	−159.20, −31.80	11120.81	10869.93, 11371.69	117.45
MDC x	−238.50	−243.40, −233.60	−497.00	−618.52, −375.48	2.08

* Change seen in all macrophage types;

# Change seen in this macrophage type only;

x Change also seen in MΦ macrophages; Cytokine levels for days 8 and 11 are shown in Supplemental Table 1; Levels for IL-8, IL-10, and TNF-α were above the detection limit at both time points and are therefore not reported

**Table 2:** Cytokines differentially secreted by M2 macrophages in response to VSL#3.

**Analyte**	**M2 (pg/mL)**		**M2 + VSL#3 (pg/mL)**		**Fold-Change**
	**Mean ΔCCS**	**95% CI**	**Mean ΔCCS**	**95% CI**	593.08
IL-1β *	0.12	−0.12, 0.36	71.17	70.92, 71.42	
IL-1Ra	187.00	159.56, 214.44	490.00	462.56, 517.44	2.62
IL-2	11.15	9.48, 12.82	0.75	−1.31, 2.81	0.07
IL-6 *	3.15	0.90, 5.40	1813.15	1621.56, 2004.74	575.60
IL-7	2.55	0.49, 4.61	62.60	51.23, 73.97	24.55
IL-8	−23.70	−49.77, 2.37	8367.00	8341.52, 8392.48	354.04
IL-9	0.60	0.50, 0.70	50.78	48.67, 52.88	84.63
IL-10	60.70	44.43, 76.97	9718.39	9701.73, 9735.05	160.11
IL-12p40	4.30	3.12, 5.48	545.15	498.01, 592.29	126.78
IL-12p70	2.90	2.90, 2.90	24.65	23.96, 25.34	8.50
IL-13	6.75	4.30, 9.20	21.15	12.82, 29.48	3.13
IL-15	9.05	3.07, 15.03	24.65	24.36, 24.94	2.72
EGF	1.35	1.25, 1.45	12.80	12.41, 13.19	9.48
Eotaxin	1.40	1.20 1.60	26.45	23.61, 29.29	18.89
FGF-2	4.75	3.67, 5.83	19.35	16.51, 22.19	4.07
Fractalkaline #	96.00	92.08, 99.92	237.50	207.12, 267.88	2.47
G-CSF *	23.60	14.19, 33.01	2134.85	2058.31, 2211.39	90.46
GRO	16.60	10.52, 22.68	4591.80	3891.49, 5292.11	276.61
IFN-α2	10.45	9.18, 11.72	39.50	37.93, 41.07	3.78
MCP-3	276.30	176.93, 375.67	45.65	45.16, 46.14	0.17
MIP-1α	20.65	13.89, 27.41	309.55	300.04, 319.06	14.99
MIP-1β	91.70	45.44, 137.96	980.80	770.30, 1191.30	10.70
TGF-α	33.00	32.22, 33.78	302.85	302.56, 303.14	9.18
TNF-α	17.15	2.35, 31.95	3378.05	3004.77, 3751.33	196.97
VEGF	65.80	56.98, 74.62	7630.30	7455.66, 7804.94	115.96

* Change seen in all macrophage types;

# Change seen in this macrophage type only; Cytokine levels for days 8 and 11 are shown in Supplemental Table 2; Levels for IL-4 were above the detection limit at both time points and are therefore not reported

**Table 3:** Cytokines differentially secreted by MΦ macrophages in response to VSL#3.

**Analyte**	**MΦ (pg/mL)**		**MΦ + VSL#3 (pg/mL)**		**Fold-Change**
	**Mean ΔCCS**	**95% CI**	**Mean ΔCCS**	**95% CI**	Undef
IL-1β *	0.00	0.00, 0.00	107.64	29.24, 186.04	
IL-1Ra	37.05	32.25, 41.85	291.55	132.30, 450.80	7.87
IL-2	4.45	4.16, 4.74	1.95	1.07, 2.83	0.44
IL-6 *	−2.20	−6.12, 1.72	2929.10	1492.81, 4365.39	1332.41
IL-7	−1.45	−3.31, 0.41	58.05	44.23, 71.87	41.03
IL-8	−163.50	−338.92, 11.92	8277.50	8192.24, 8362.76	51.63
IL-9	0.05	0.05, 0.05	3.20	1.93, 4.47	64.00
IL-10	8.85	6.60, 11.10	9727.89	9702.41, 9753.37	1099.20
IL-12p40	2.55	0.30, 4.80	465.45	38.66, 892.24	182.53
IL-12p70	1.10	0.90, 1.30	10.05	6.03, 14.07	9.14
IL-13	0.95	0.85, 1.05	4.70	1.76, 7.64	4.95
IL-15	5.90	4.53, 7.27	19.55	14.94, 24.16	3.31
EGF	−0.35	−2.60, 1.90	12.17	8.50, 15.84	35.77
Eotaxin	1.35	0.27, 2.43	27.05	21.86, 32.24	20.04
FGF-2	1.35	−0.12, 2.82	13.45	10.80, 16.10	9.96
G-CSF *	7.15	2.54, 11.76	8030.45	1601.16, 14459.74	1123.14
GM-CSF #	5.20	−0.68, 11.08	83.55	30.34, 136.76	16.07
GRO	28.30	28.10, 28.50	4738.70	3277.52, 6199.88	167.45
IFN-α2	7.35	5.10, 9.60	26.40	25.81, 26.99	3.59
IFN-γ #	2.10	0.92, 3.28	102.70	32.34, 173.06	48.90
MCP-1 #	257.00	241.32, 272.68	−8020.50	−10885.04, −5155.96	32.21
MCP-3	40.80	32.18, 49.42	10.45	−1.60, 22.50	0.26
MDC x	5001.50	4822.16, 5180.84	1046.50	590.80, 1502.20	0.21
MIP-1α	20.25	14.86, 25.64	242.70	78.45, 406.95	11.99
MIP-1β	20.40	11.58, 29.22	140.80	75.14, 206.46	6.90
TGF-α	4.80	4.41, 5.19	34.90	21.18, 48.62	7.27
TNF-α	2.50	2.30, 2.70	1539.65	552.30, 2527.00	615.86
VEGF	13.15	11.29, 15.01	6746.25	3471.38, 10021.12	513.02

* Change seen in all macrophage types;

# Change seen in this macrophage type only;

x Change also seen in M1 macrophages; Undef = undefined; Cytokine levels for days 8 and 11 are shown in Supplemental Table 3

## Abbreviations

ANOVA: Analysis of Variance
ΔCCS: Change in Cytokine and Chemokine Secretion
CD: Crohn’s Disease
CD#: Cluster of Differentiation
CFU: Colony Forming Units
CXCL#: Chemokine with two cysteines separated by an amino acid on the N-terminus
EDTA: Ethylenediamidetetraacetic acid
EGF: Epidermal Growth Factor
FAO: Food and Agriculture Organization of the United Nations
FBS: Fetal Bovine Serum
FGF-2: Fibroblast Growth Factor 2
FITC: Fluorescein Isothiocyanate
G-CSF: Granulocyte Colony Stimulating Factor
GM-CSF: Granulocyte-Monocyte Colony Stimulating Factor
GRO: Growth-Related Oncogene
IBD: Inflammatory Bowel Disease
IFN-α2: Interferon alpha 2
IFN-γ: Interferon gamma
IgG: Immunoglobulin G
IL-1α: Interleukin 1 alpha
IL-1β: Interleukin 1 beta
IL-1Ra: Interleukin 1 receptor antagonist
IL-2: Interleukin 2
IL-4: Interleukin 4
IL-6: Interleukin 6
IL-7: Interleukin 7
IL-8: Interleukin 8, also known as CXCL8
IL-9: Interleukin 9
IL-10: Interleukin 10
IL-12p40: Interleukin 12 p40 subunit
IL-12p70: Interleukin 12
IL-13: Interleukin 13
IL-15: Interleukin 15
IL-17A: Interleukin 17A
IL-23: Interleukin 23
LPS: Lipopolysaccharide
M1: Classically activated or proinflammatory macrophage
M2: Alternatively activated or anti-inflammatory macrophage
M2b: Regulatory, or type II, M2 macrophage
MΦ: Unpolarized macrophage
MCP-1: Macrophage Chemoattractant Protein 1, also known as CCL2
MCP-3: Macrophage Chemoattractant Protein 3, also known as CCL7
M-CSF: Macrophage Colony Stimulating Factor
MDC: Macrophage-Derived Chemokine, also known as CCL22
MIP-1α: Macrophage Inhibitory Protein 1 alpha
MIP-1β: Macrophage Inhibitory Protein 1 beta
NF-κB: Nuclear Factor kappa B
PBMC: Peripheral Blood Mononuclear Cell
PBS: Phosphate Buffered Saline
PerCP: Peridinin Chlorophyll
SEM: Standard Error of the Mean
Th1: Type 1 helper T cell
Th2: Type 2 helper T cell
TGF-α: Transforming Growth Factor alpha
TNF-α: Tumor Necrosis Factor alpha
UC: Ulcerative Colitis
VEGF: Vascular Endothelial Growth Factor
WHO: World Health Organization
