# Supplementary material for: The Probiotic Mixture VSL#3 Alters the Morphology and Secretion Profile of Both Polarized and Unpolarized Human Macrophages in a Polarization-Dependent Manner
Source: J Clin Cell Immunol. Author manuscript; Available in PMC 2014 Aug 27. (PMC4145411; doi:10.4172/2155-9899.1000227)
Supplement: All supplementary tables [file NIHMS615061-supplement-All_supplementary_tables.docx]

**Supplemental Material**

| **Supplemental Table 1.** Mean levels at days 8 and 11 for cytokines differentially secreted by M1 macrophages in response to VSL#3 | | | | |
| --- | --- | --- | --- | --- |
| Analyte | Day 8 (mean ± SEM; pg/mL) | | Day 11 (mean ± SEM; pg/mL) | |
|  | M1 | M1 + VSL#3 | M1 | M1 + VSL#3 |
| IL-1α | 28.10 ± 5.60 | 21.95 ± 1.55 | 27.7 ± 5.60 | 35.85 ± 0.45 |
| IL-1β | 121.95 ± 53.05 | 76.10 ±13.20 | 82.00 ± 37.00 | 401.0 ± 11.00 |
| IL-4 | 22.85 ± 0.25 | 22.00 ± 2.20 | 18.90 ± 0.10 | 30.4 ± 0.10 |
| IL-6 | 5829.76 ± 4282.76 | 4210 ± 1030.00 | 4769.00 ± 3694.00 | 10112.52 ± 0.00 |
| IL-17A | 4.85 ± 0.75 | 10.25± 1.35 | 3.05 ± 0.05 | 6.20 ± 1.20 |
| G-CSF | 732 ± 379.00 | 537 ± 128.00 | 18.90 ± 0.10 | 30.40 ± 0.10 |
| MDC | 1073 ± 47.00 | 1507.00 ± 62.00 | 834.5 ± 49.50 | 1010.00 ± 0.00 |
|  | | | | |

| **Supplemental Table 2.**  Mean levels at days 8 and 11 for cytokines differentially secreted by M2 macrophages in response to VSL#3 | | | | |
| --- | --- | --- | --- | --- |
| Analyte | Day 8 (mean ± SEM; pg/mL) | | Day 11 (mean ± SEM; pg/mL) | |
|  | M2 | M2 + VSL#3 | M2 | M2 + VSL#3 |
| IL-1β | 1.36 ± 0.00 | 1.38 ± 0.02 | 1.48 ± 0.12 | 72.55 ± 0.15 |
| IL-1Ra | 123.5 ± 3.50 | 132.50 ± 16.50 | 310.50 ± 17.50 | 622.50 ± 2.50 |
| IL-2 | 2.90 ± 0.10 | 4.20 ± 0.40 | 14.05 ± 0.95 | 4.95 ± 0.65 |
| IL-6 | 6.90 ± 0.80 | 12.85 ± 1.25 | 10.05 ± 1.95 | 1826.00 ± 99.00 |
| IL-7 | 8.35 ± 0.55 | 9.30 ± 0.50 | 2.55 ± 1.05 | 62.60 ± 5.80 |
| IL-8 | 148.50 ± 40.50 | 300.00 ± 13.00 | 124.80 ± 27.20 | 8667.00 ± 0.00 |
| IL-9 | 1.45 ± 0.00 | 1.48 ± 0.03 | 2.05 ± 0.05 | 52.25 ± 1.05 |
| IL-10 | 106.30 ± 10.70 | 123.50 ± 8.50 | 167.00 ± 19.00 | 9841.89 ± 0.00 |
| IL-12p40 | 8.10 ± 0.00 | 7.35 ± 0.55 | 12.40 ± 0.60 | 552.50 ± 23.50 |
| IL-12p70 | 3.80 ± 0.00 | 3.10 ± 0.10 | 6.70 ± 0.00 | 27.75 ± 0.25 |
| IL-13 | 3.80 ± 0.30 | 4.15 ± 0.65 | 10.55 ± 1.55 | 25.30 ± 4.90 |
| IL-15 | 3.95 ± 0.45 | 4.60 ± 0.40 | 13.00 ± 3.50 | 29.25 ± 0.55 |
| EGF | 5.60 ± 0.70 | 6.05 ± 1.15 | 6.95 ± 0.75 | 18.85 ± 0.95 |
| Eotaxin | 10.75 ± 0.85 | 12.35 ± 1.05 | 12.15 ± 0.95 | 38.80 ± 0.40 |
| FGF-2 | 14.95 ± 0.85 | 12.90 ± 0.20 | 19.70 ± 0.30 | 32.25 ± 1.25 |
| Fractalkaline | 135.50 ± 21.50 | 146.00 ± 0.00 | 231.50 ± 19.50 | 383.50 ± 15.50 |
| G-CSF | 46.70 ± 0.30 | 49.15 ± 3.05 | 70.30 ± 4.50 | 2184.00 ± 36.00 |
| GRO | 29.80 ± 1.40 | 30.20 ± 3.70 | 46.40 ± 1.70 | 4622.00 ± 361.00 |
| IFN-α2 | 13.35 ± 0.75 | 12.65 ± 0.25 | 23.80 ± 0.10 | 52.15 ± 0.55 |
| MCP-3 | 65.20 ± 4.80 | 75.35 ± 14.75 | 341.50 ± 55.50 | 121.00 ± 15.00 |
| MIP-1α | 32.40 ± 2.90 | 37.95 ± 5.35 | 53.05 ± 0.55 | 347.50 ± 0.50 |
| MIP-1β | 75.80 ± 3.90 | 89.2 ± 10.40 | 167.50 ± 27.50 | 1070.00 ± 97.00 |
| TGF-α | 6.65 ± 0.05 | 8.15 ± 0.85 | 39.65 ± 0.45 | 311.00 ± 1.00 |
| TNF-α | 16.25 ± 1.35 | 24.45 ± 0.95 | 33.40 ± 8.90 | 3402.50 ± 189.50 |
| VEGF | 41.70 ± 1.00 | 40.20 ± 8.60 | 107.50 ± 5.50 | 7670.50 ± 80.50 |
|  | | | | |

| **Supplemental Table 3.** Mean levels at days 8 and 11 for cytokines differentially secreted by MΦ macrophages in response to VSL#3 | | | | |  |
| --- | --- | --- | --- | --- | --- |
| Analyte | Day 8 (mean ± SEM; pg/mL) | | Day 11 (mean ± SEM; pg/mL) | | |
|  | MΦ | MΦ + VSL#3 | MΦ | MΦ + VSL#3 | |
| IL-1β | 1.36 ± 0.00 | 1.36 ± 0.00 | 1.36 ± 0.00 | 109.00 ± 40.00 | |
| IL-1Ra | 60.15 ± 9.35 | 58.45 ± 4.25 | 97.20 ± 11.80 | 350.00 ± 77.00 | |
| IL-2 | 3.00 ± 0.30 | 2.80 ± 0.10 | 7.45 ± 0.15 | 4.75 ± 0.35 | |
| IL-6 | 13.25 ± 7.85 | 5.90 ± 2.80 | 11.05 ± 5.85 | 2935.00 ± 7.30 | |
| IL-7 | 13.25 ± 3.05 | 9.90 ±1.50 | 11.80 ± 2.10 | 67.95 ± 5.55 | |
| IL-8 | 540.5 ± 168.50 | 389.50 ± 43.50 | 377.00 ± 79.00 | 8667.00 ± 0.00 | |
| IL-9 | 1.45 ± 0.00 | 1.45 ± 0.00 | 1.50 ± 0.00 | 4.65 ± 0.65 | |
| IL-10 | 98.60 ± 10.40 | 114 ± 13.00 | 107.45 ± 11.55 | 9841.89 ± 0..00 | |
| IL-12p40 | 7.50 ± 1.50 | 6.55 ± 0.75 | 10.05 ± 0.35 | 472.00 ± 217.00 | |
| IL-12p70 | 3.30 ± 0.30 | 3.25 ± 0.55 | 4.40 ± 0.40 | 13.30 ± 1.50 | |
| IL-13 | 3.20 ± 0.10 | 3.20 ± 0.40 | 4.15 ± 0.05 | 7.90 ± 1.10 | |
| IL-15 | 4.00 ± 0.00 | 3.70 ± 0.40 | 9.90 ± 0.70 | 23.25 ± 2.75 | |
| EGF | 6.10 ± 1.20 | 3.68 ± 0.62 | 5.75 ± 0.05 | 15.85 ± 1.25 | |
| Eotaxin | 13.75 ± 1.95 | 11.55 ± 2.35 | 15.10 ± 1.40 | 38.60 ± 0.30 | |
| FGF-2 | 14.70 ± 2.00 | 12.30 ± 1.10 | 16.05 ± 1.25 | 25.75 ± 0.25 | |
| G-CSF | 15.65 ± 3.15 | 12.55 ± 1.25 | 22.80 ± 0.80 | 8043.00 ± 3279.00 | |
| GM-CSF | 7.75 ± 1.25 | 5.80 ± 0.50 | 12.95 ± 4.25 | 89.35 ± 27.65 | |
| GRO | 40.85 ± 12.45 | 33.30 ± 10.50 | 69.15 ± 12.55 | 4772 ± 735.00 | |
| IFN-α2 | 11.05 ± 1.35 | 10.50 ± 1.30 | 18.40 ± 0.20 | 36.90 ± 1.00 | |
| IFN-γ | 3.30 ± 0.90 | 2.75 ± 0.35 | 5.40 ± 0.30 | 105.45 ± 35.55 | |
| MCP-1 | 9821.00 ± 291.00 | 8987.00 ± 1550.00 | 10078.00 ± 283.00 | 966.50 ± 88.50 | |
| MCP-3 | 40.85 ± 13.55 | 26.80 ± 3.00 | 81.65 ± 9.15 | 37.25 ± 3.15 | |
| MDC | 1561.50 ± 161.50 | 1552.50 ±153.50 | 6563.00 ± 253.00 | 2599.00 ± 386.00 | |
| MIP-1α | 23.65 ± 5.25 | 17.30 ± 1.80 | 43.90 ± 2.50 | 260.00 ± 82.00 | |
| MIP-1β | 33.35 ± 4.05 | 29.20 ± 5.50 | 53.75 ± 8.55 | 170.00 ± 28.00 | |
| TGF-α | 1.10 ± 0.60 | 0.40 ± 0.00 | 5.90 ± 0.80 | 35.30 ± 7.00 | |
| TNF-α | 16.90 ± 4.70 | 12.85 ± 3.25 | 19.40 ± 4.60 | 1552.50 ± 500.50 | |
| VEGF | 31.60 ± 3.00 | 25.75 ± 6.85 | 44.75 ± 2.05 | 6772.00 ± 1664.00 | |
|  | | | | |  |
